# Long Lasting Research on the Atomically Precise Au_144_(SR)_60_ Nanocluster

**DOI:** 10.1021/acscentsci.5c00988

**Published:** 2025-07-14

**Authors:** Rongchao Jin

**Affiliations:** Department of Chemistry, Carnegie Mellon University, Pittsburgh, Pennsylvania 15213, United States

## Abstract

Magnetic circular dichroism spectroscopy reveals the intricate electronic structure of Au_144_(SCH_2_CH_2_Ph)_60_ .

## Graphical abstract

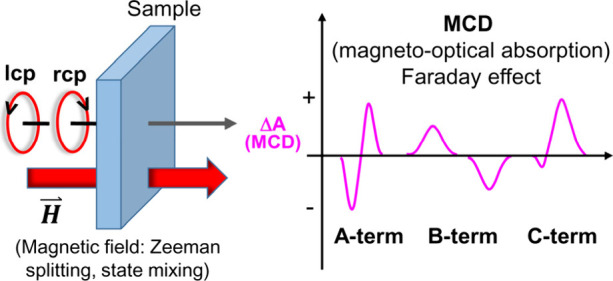

Atomically precise gold nanoclusters emerged from nanoscience research in the late 2000s.^1^ These nanoclusters are unique in that they possess precise molecular formulas (i.e., specific numbers of gold atoms and surface ligands), atomic total structures, and novel properties such as quantized electronic structures, tunable energy gaps (*E*_g_) ranging from ∼2.5 electronvolt (eV) to ∼zero, and superatomic magnetism.^1^ Among them, the 144-atom Au_144_(SR)_60_ nanocluster (where SR represents thiolate ligands) has attracted attention since its isolation.^1^ However, the correlation between its structure and properties is not yet fully understood, especially its electronic structure, which is of paramount importance in order to fully realize its application in optoelectronics and quantum technology.

In this issue of *ACS Central Science*, Knappenberger and co-workers introduced a spectroscopic technique, called magnetic circular dichroism (MCD) or magneto-optical absorption, to probe Au_144_(SCH_2_CH_2_Ph)_60_.

Their results provide a new perspective on the intricate electronic structure of this long-lasting, magic-size nanocluster.^2^

In the late 1990s, Alvarez et al. identified a nanogold species with a mass of ∼29 kDa and a narrow size distribution (see review in ref 1), but no atomic precision was attained at that time. More than a decade later, in 2008, Chaki et al. successfully isolated and characterized the atomically precise Au_144_(S-*n*-C_12_H_25_)_59_ nanocluster.^3^ Subsequently, in 2009, Qian et al. devised a “one pot, one size” size-focusing synthetic method with high yield and corrected the formula to Au_144_(SR)_60_.^4^ By then, the atomically precise Au_144_(SR)_60_ nanocluster was established (Figure 1A),^1^ but the determination of its atomic structure took another decade, despite tremendous effort by many research groups! It should be mentioned that in 2016, Zeng et al. successfully crystallized a larger nanocluster, Au_246_(S-Ph-*p*-CH_3_)_80_, and identified the critical roles of ligand–ligand interactions in the crystallization of large nanoclusters.^5^ Inspired by Zeng et al.’s success, Yan et al. designed a −CH_2_– shorter ligand, i.e., −SCH_2_Ph (as opposed to −SCH_2_CH_2_Ph), and in 2018 successfully crystallized Au_144_(SCH_2_Ph)_60_.^6^ Its structure was revealed to be icosahedral (Figure 1B).^6^ This nanocluster is chiral, and recent work by Truttmann et al. has led to the attainment of pure enantiomers.^7^

**Figure 1:**
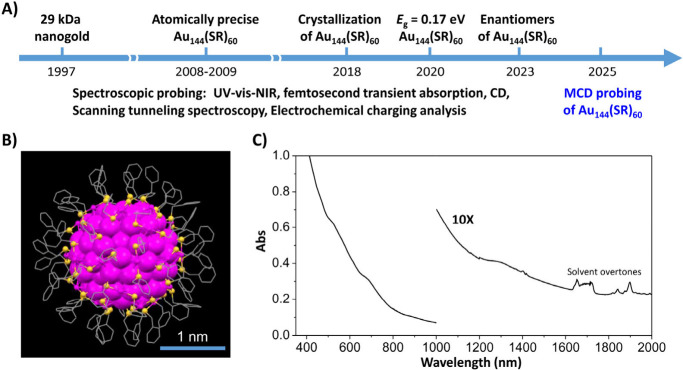
(A) Historical timeline of research on Au_144_(SR)_60_; (B) atomic structure of Au_144_(SCH_2_Ph)_60_ determined by X-ray crystallography (magenta, Au; yellow, S; and gray, carbon tails); and (C) its UV–vis–NIR absorption spectrum (the 1000–2000 nm region is enlarged 10-fold). Panel B redrawn from ref 6. Available under a CC BY-NC license. Copyright 2018 Nan Yan, Nan Xia, Lingwen Liao, Min Zhu, Fengming Jin, Rongchao Jin, and Zhikun Wu. Panel C redrawn from ref 1. Copyright 2016 American Chemical Society.

Although the atomic structure of Au_144_(SR)_60_ was successfully determined in 2018, fully understanding its electronic and optical properties requires considerable effort. The optical absorption of Au_144_(SR)_60_ spans the ultraviolet–visible–near-infrared (UV–vis–NIR) range (Figure 1C) and even extends into the infrared. In early research, Au_144_(SR)_60_ was thought to be in a metallic state based on its ultrafast electron relaxation time (a few picoseconds, comparable to that of the metallic state). However, later studies contradicted this, particularly a meticulous electrochemical single-electron charging analysis by Wang and co-workers in 2020, which determined an accurate HOMO–LUMO gap (*E*_g_ = 0.17 eV).^8^ Thus, Au_144_(SR)_60_ is nonmetallic.

While earlier reports had provided some understanding of the electronic properties of Au_144_(SR)_60_,^1,3,4,6–8^ many details remained unknown. In this regard, Knappenberger and co-workers introduced the MCD technique.^2^ To appreciate their work, one needs to understand some basics about MCD. Let us start with light polarization, which refers to the electric field ( $\overset{⇀}{E}$ ) state: oscillating vertically/horizontally (called linear polarization), or rotating clockwise/counterclockwise (called the right-handed circular polarization (rcp) and lcp, respectively). According to quantum mechanics, lcp or rcp light carries a spin angular momentum of one unit, *h*/2π (note: polarization is understood as spin in quantum theory), and this momentum is transferred to the molecule during the absorption. The difference in absorption (*A*) of lcp and rcp light by a substance is defined as circular dichroism (CD), CD = Δ*A* = *A*_lcp_ – *A*_rcp_. Regular CD spectroscopy can only probe chiral samples since their structures exhibit helicity or bear chiral carbon centers.

MCD spectroscopy does not require chiral structures, because the magnetic field ( $\overset{⇀}{H}$ ) in the MCD spectrometer can induce the CD signals (Figure 2). An MCD spectrum may contain up to three types of signals, referred to as Faraday A-, B-, and C-terms (Figure 2). Among them, the A- and B-term signals are temperature independent and their intensities are linearly proportional to the field strength, while the C-term is temperature dependent and becomes stronger at cryogenic temperatures and saturates at sufficiently strong magnetic field strengths.

**Figure 2:**
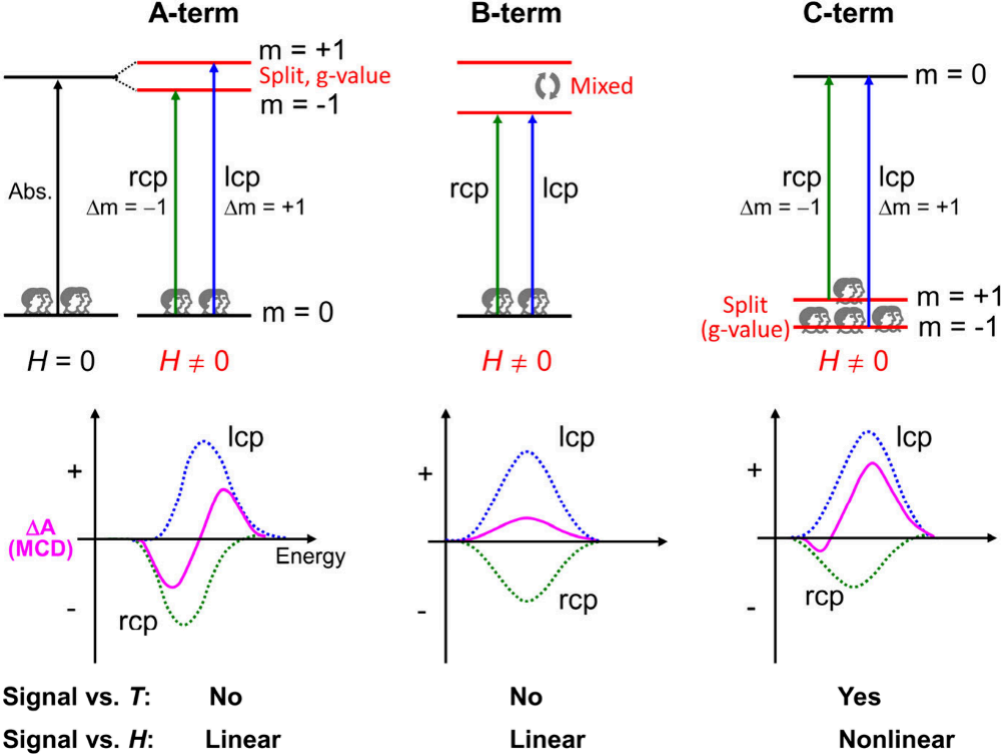
Origins of the three types of MCD signals: Faraday A-term, B-term, and C-term (see descriptions in main text). The heads represent initial populations on the states prior to electronic MCD transitions (selection rule: the magnetic quantum number change, Δ*m* = −1, for rcp light absorption, and Δ*m* = +1 for lcp light absorption). The *m* = 0 level is omitted.

With the above background, we can now appreciate Knappenberger and co-workers’ MCD analysis of Au_144_(SCH_2_CH_2_Ph)_60_.^2^ Our first reaction is—wow, so many peaks! Even within the narrow range of 500–730 nm (c.f., the ordinary absorption in Figure 1C), there are already 11 distinct MCD peaks (counting positive and negative ones).

Such a fine structure reflects the dense states and complex electronic structure of Au_**144**_ **(SR)**_**60**_.

Briefly, based upon the variable-temperature, variable $\overset{⇀}{H}$ field (VTVH) analysis, Foxley et al. divided the MCD spectrum of Au_144_(SR)_60_ into two distinct regimes: Region I, 1.7–2.0 eV (730–620 nm), and Region II, 2.0–2.5 eV (620–500 nm).^2^ In Region I, various MCD peak behaviors were observed, including B- and C-term signals (or the B/C mix), such as a B-term at 1.85 eV and a C-term at 1.8 eV. By fitting the trend of each MCD signal with theory, one can determine the *g*-factors of the relevant excited, ground, or mixed states (see red highlighted states in Figure 2). For example, Figure 3 shows the 1.85 eV B-term signal (a linear trend) and 1.8 eV C-term signal (nonlinear rising and then saturated at high fields). The 1.9 eV MCD signal is a mix of B- and C-terms, as it shows both linear and saturating behaviors, and data fitting gives a large *g*-value of 4.4 ± 1.1, indicating a large angular momentum (orbital and/or spin) for the ground state. The observed paramagnetic behavior at 1.8 and 1.9 eV, respectively, suggests their origins in the unpaired electrons in the superatomic HOMO manifold of Au_144_(SCH_2_CH_2_Ph)_60_.^2^ In Region II, excitations to ligand vibronic states involving S–C vibrations were identified.^2^

**Figure 3:**
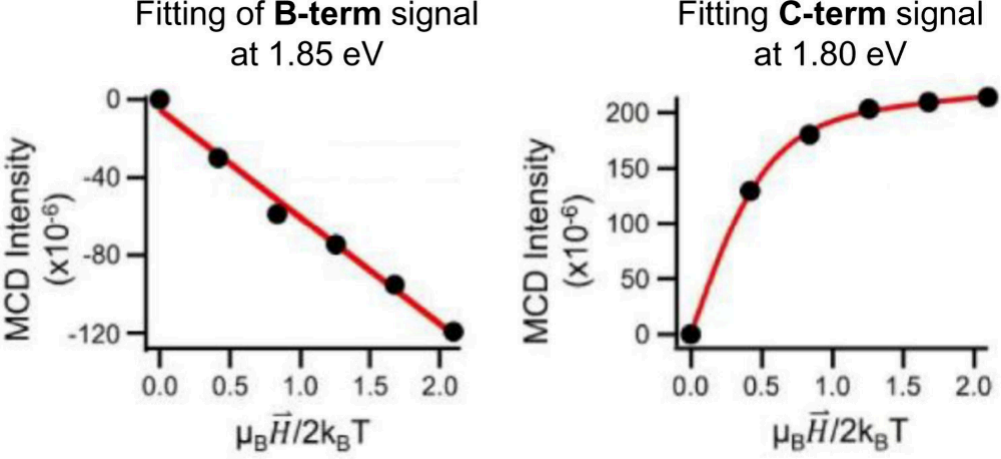
Illustration of MCD signals from Au_144_(SCH_2_CH_2_Ph)_60_, including a B-term signal (negative) at 1.85 eV and its fitting (red line) with theory, and a C-term signal (positive) at 1.8 eV and its fitting (red). Reproduced from ref 2. Available under a CC-BY 4.0 license. Copyright 2025 Juniper Foxley, Marcus Tofanelli, Jane A. Knappenberger, Christopher J. Ackerson, Kenneth L. Knappenberger Jr.

Overall, this fine MCD spectroscopic study reveals many interesting features of the intricate electronic properties of the Au_144_(SR)_60_ nanocluster by assessing its electronic orbital, spin, and magnetic properties.

Given that the UV–vis–NIR spectrum of Au_144_(SR)_60_ spans a broad range from the UV to the IR, future MCD probing in the NIR region is expected to yield further insights.

We aspire to construct a complete, grand orbital picture of the Au_144_(SR)_60_ and explore the evolution from discrete orbitals to band structure in larger Au_*n*_(SR)_*m*_ nanoclusters,^9^ along with their nascent plasmon properties as probed by MCD.
